# Fecal *Klebsiella pneumoniae* Carriage Is Intermittent and of High Clonal Diversity

**DOI:** 10.3389/fmicb.2020.581081

**Published:** 2020-11-24

**Authors:** Sarah Lepuschitz, Kathrin Hauser, Agnes Schriebl, Claudia Schlagenhaufen, Anna Stöger, Ali Chakeri, Kornelia Vötsch, Shiva Pekard-Amenitsch, Burkhard Springer, Franz Allerberger, Werner Ruppitsch

**Affiliations:** Austrian Agency for Health and Food Safety, Institute of Medical Microbiology and Hygiene, Graz, Austria

**Keywords:** *Klebsiella pneumoniae*, long-term study, colonization, whole genome sequencing, virulence, antimicrobial resistance

## Abstract

The *Klebsiella pneumoniae* complex comprises several closely related entities, which are ubiquitous in the natural environment, including in plants, animals, and humans. *K. pneumoniae* is the major species within this complex. *K. pneumoniae* strains are opportunistic pathogens and a common cause of healthcare-associated infections. *K. pneumoniae* can colonize the human gastrointestinal tract, which may become a reservoir for infection. The aim of this study was to investigate the fecal *K. pneumoniae* carriage in six healthy individuals during a 1 year period. Stool samples were obtained once a week. Using direct and pre-enriched cultures streaked on ampicillin-supplemented agar plates, up to eight individual colonies per positive sample were selected for further characterization. Whole genome sequencing (WGS) was performed for strain characterization. Sequence type (ST), core genome complex type (CT), K and O serotypes, virulence traits, antibiotic resistance profiles, and plasmids were extracted from WGS data. In total, 80 *K. pneumoniae* isolates were obtained from 48 positive cultures of 278 stool samples from five of the six test subjects. The samples of the five colonized volunteers yielded at most two, three, four (two persons), and five different strains, respectively. These 80 *K. pneumoniae* isolates belonged to 60 STs, including nine new STs; they were of 70 CTs, yielded 48 K serotypes, 11 O serotypes, and 39 *wzc* and 51 *wzi* alleles. Four of the five subjects harbored serotypes K20 and K47, as well as STs ST37, ST101, ST1265, and ST20, which had previously been linked to high-risk *K. pneumoniae* clones. In total, 25 genes conferring antibiotic resistance and 42 virulence genes were detected among all 80 isolates. Plasmids of 15 different types were found among 65 of the isolates. Fecal carriage of individual strains was of short duration: 70 strains were found on a single sampling day only, and 5 strains were isolated in samples collected over two consecutive weeks. Two of the five colonized individuals—working colleagues having meals together—shared identical *K. pneumoniae* types four times during the study period. Our findings point toward the potential role of food as a reservoir for *K. pneumoniae* in humans.

## Introduction

*Klebsiella pneumoniae* was first described in 1882 as a bacterium isolated from the lungs of patients who had died from pneumonia (Friedlaender, 1882). The *K. pneumoniae* complex consists of closely related species designated as *K. pneumoniae* phylogroups Kp1-Kp7, comprising *K. pneumoniae* subsp. *ozaenae, K. pneumoniae* subsp. *pneumoniae, K. pneumoniae* subsp. *rhinoscleromatis, K. quasipneumoniae* subsp. *quasipneumoniae*, *K. quasipneumoniae* subsp. *similipneumoniae*, *K. variicola* subsp. *variicola*, *K. variicola* subsp. *tropica*, *K. africana*, and *K. quasivariicola* (Rodrigues et al., 2018, 2019). *K. pneumoniae* complex can be found ubiquitously in nature, including in plants, animals, and humans (Lai et al., 2019). Most *K. pneumoniae* infections in Europe and North America are healthcare-associated and caused by classical *K. pneumoniae* strains (cKp) (Russo et al., 2018). With the emergence of carbapenem-resistant strains, infections due to cKp have become a major public health threat (World Health Organization [WHO], 2017; Wyres and Holt, 2018) causing life-threatening nosocomial infections like urinary tract infections, bloodstream infections, and pneumonia in immunocompromised and critically ill patients (Podschun and Ullmann, 1998). *K. pneumoniae* is a listed ESKAPE pathogen, an acronym defined by the Infectious Diseases Society of America for antibiotic-resistant *Enterococcus faecium*, *Staphylococcus aureus, K. pneumoniae, Acinetobacter baumannii, Pseudomonas aeruginosa*, and *Enterobacter* spp. (Rice, 2008). In 1986, hypervirulent *K. pneumonia* (hvKp) strains emerged in Asian countries associated with community-acquired infections like pyogenic liver abscess, meningitis, endophthalmitis, soft tissue abscesses, urinary tract infections, and pneumonia (Martin and Bachman, 2018; Russo and Marr, 2019). In contrast to cKp strains, hvKp strains cause infections mainly in young and healthy individuals (Struve et al., 2015; Paczosa and Mecsas, 2016). In contrast to cKp, which is the dominating cause of infections in Western countries, hvKp are endemic mainly in countries of the Asia-Pacific region. A differentiation between cKp and hvKp is challenging due to overlapping characteristics in both pathotypes (Russo and Marr, 2019). Several virulence factors present on large virulence plasmids (pK2044 and pLVPK) have been identified, allowing the most accurate discrimination of cKp to hvKp (Lee et al., 2017; Russo et al., 2018; Russo and Marr, 2019). Key virulence factors necessary for infection are the polysaccharide capsule (K antigen) and lipopolysaccharide (O antigen), which contribute to serum resistance and resistance to phagocytosis (Cortés et al., 2002). HvKp clones circulating in the community are associated with particular capsule types, mainly K1, K2, K20, and K57 (Lee et al., 2016) and certain sequence types (STs) like ST23, ST65, ST86, ST375, and ST380 (Bialek-Davenet et al., 2014; Lin et al., 2014; Lee et al., 2017). The convergence of carbapenem-resistance and virulence resulted in the emergence of carbapenem-resistant hvKP strains in China, which is expected to become a serious future public health issue (Zhao et al., 2020).

*K. pneumoniae* can colonize the nasopharynx and the gastrointestinal tract. The gastrointestinal colonization of healthy individuals with undefined pathotypes ranged from 5 to 35% in Western countries (Martin et al., 2016; Gorrie et al., 2017) and from 19 to 88% in Asian countries (Chung et al., 2012). Nasopharyngeal colonization of healthy humans ranged from 1 to 5% in Western countries and from 1.4 to >20% in Asian countries and Brazil (Lima et al., 2010; Farida et al., 2013; Dao et al., 2014). Contamination of food with *K. pneumoniae* and a general poor sanitation status have been associated with increased colonization of healthy humans (Farida et al., 2013; Huynh et al., 2020). In a study from Malaysia, 32% of street food samples tested positive for *K. pneumoniae* (Haryani et al., 2007). Colonization has been identified as a potential reservoir for infection with Kp strains (Gorrie et al., 2017) and the infection risk with *K. pneumoniae* is considered to be four times higher for colonized patients compared to non-carriers (Selden et al., 1971; Martin et al., 2016). During warm months, *K. pneumoniae* bloodstream infection rates are 1.5 times higher, reflecting an increased fecal carriage rate in humans in summer (Anderson et al., 2008). Therefore, screening of healthy individuals is a recommended action to obtain an overview on strain diversity and to detect emerging resistant and virulent strains (Russo and Marr, 2019).

To our best knowledge, there is no longitudinal Kp colonization study of healthy individuals. Most studies are focused on short/long-term colonization of hospitalized patients. Therefore, the aim of this study was to investigate the colonization pattern of *K. pneumoniae* in healthy humans during a 1 year period.

## Materials and Methods

### Sample Collection and Microbiological Culturing of *K. pneumoniae*

From calendar week (CW) 15/2018 to CW14/2019, fecal samples from six healthy individuals were screened for the presence of *K. pneumoniae*. Fecal samples of about 2 g were collected in sterile plastic containers once a week and processed in the laboratory within 24 h. Volunteers lived in six different households in Vienna (subject 1) and Graz (subjects 2–6). Subjects 2 and 4 often spent lunch breaks together, having their meals in various restaurants. Subject 1 was 60–65 years old, subjects 2 and 4 were aged 25–30, subject 3 was aged 40–45, and subjects 5 and 6 were aged 50–55 years. Subject 4 followed a gluten-free diet. Subject 6 was vegetarian but ate fish.

To detect *K. pneumoniae*, all feces samples were plated on Simmons Citrate Agar with 1% Innositol (SCAI) (BIO-RAD, Hercules, United States) and incubated for 48 h at 44°C. In addition, broth enrichment was performed (1 g feces in 9 ml LB medium with 10 μg/l ampicillin overnight at 37°C), followed by cultivation on an SCAI medium for 48 h at 44°C. Up to eight single colonies resembling *K. pneumoniae* morphologically were selected from each agar plate and subcultured for further processing. Species confirmation was carried out using matrix assisted laser desorption/ionization time-of-flight mass spectrometry (MALDI-TOF-MS) Biotyper (Bruker, Billerica, MA, United States) according to the manufacturer’s instructions.

### Antimicrobial Resistance Testing

A selection of *K. pneumoniae* strains were forwarded to ESBL confirmatory testing using cefotaxime 30 μg (CTX) and cefotaxime 30 μg with clavulanic acid 10 μg (CTX-CV) disks as well as ceftazidime 30 μg (CAZ) and ceftazidime 30 μg with clavulanic acid 10 μg (CAZ-CV) disks (Mast Group, Bootle Merseyside, United Kingdom). The inhibition zone diameters were measured and assessed according to EUCAST guidelines (EUCAST, 2017).

A second plate was used for agar diffusion test with ceftazidime 10 μg (CAZ), cefotaxime 5 μg (CTX), ceftriaxone 30 μg (CRO), and amoxicilline-clavulanic acid 20/10 μg (AMC), and the inhibition zone diameters were assessed according to EUCAST criteria (EUCAST, 2020).

### DNA Extraction and Whole Genome Sequencing

DNA was isolated from bacterial cultures using the MagAttract HMW DNA Kit (Qiagen, Hilden, Germany) according to the manufacturer’s protocol for gram-negative bacteria. The amount of input DNA was quantified on a Lunatic instrument (Unchained Labs, Pleasanton, CA, United States). Ready to sequence libraries were prepared using Nextera XT DNA library preparation kit (Illumina, San Diego, CA, United States); paired-end sequencing with a read length of 2 × 300 bp using Reagent Kit v3 chemistry (Illumina) was performed on a Miseq instrument (Illumina).

### Sequence Data Analysis

All study isolates were sequenced to obtain a coverage of at least 80-fold. Obtained raw reads were quality controlled using FastQC v0.11.9 and *de novo* assembled using SPAdes (version 3.9.0) (Bankevich et al., 2012) to produce draft genomes. Contigs were filtered for a minimum coverage of 5 × and a minimum length of 200 bp using SeqSphere + software v6.0.0) (Ridom, Münster, Germany). The classical multilocus sequence type (MLST) (Diancourt et al., 2005) and the public *K. pneumoniae sensu lato* core genome MLST (cgMLST^1^) were determined using SeqSphere+. For MLST, new combinations of alleles or new allele types composing new sequence types (STs) were submitted to the curators of the MLST database^2^. For phylogenetic analysis, minimum spanning trees (MSTs) were calculated based on the *sensu lato* cgMLST scheme; related isolates were identified with a complex type (CT) distance of 15 alleles (see footnote 1).

The diversity of capsule synthesis loci (K loci), lipopolysaccharide O antigen (O loci), and allele diversity of K locus genes *wzc* and *wzi* were determined using Kaptive Web^3^ (Wick et al., 2018).

Plasmids and genes conferring antibiotic resistance were detected using PlasmidFinder 1.3 (Carattoli et al., 2014) available from the Center for Genomic Epidemiology^4^ and the comprehensive antibiotic resistance database (CARD) (Jia et al., 2017). Virulence genes were detected using the virulence allele library from the Institut Pasteur BIGSdb database for *K. pneumoniae*^5^ (Bialek-Davenet et al., 2014).

### Nucleotide Sequence Accession Numbers

This Whole Genome Shotgun project has been deposited at the DDBJ/EMBL/GenBank under the accession PRJNA663884. The version described in this paper is the first version. The raw sequence reads have been deposited in the Sequence Read Archive (SRA) under accession no. SRR12653693–SRR12653772.

## Results

**FIGURE 1:**
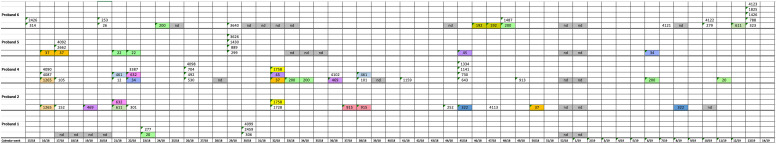
Schematic representation of *K. pneumoniae* isolation from stool samples of five test persons during the 1 year study period. Numbers show a successful isolation and represent the sequence type. Colors show isolates with the same sequence types. nd, no samples analyzed.

During the study period from CW15 in 2018 to CW14 in 2019, a total of 278 stool samples (43–49 samples/patient) were analyzed from the six study participants (Figure 1). Forty-eight of these 278 stool samples yielded *K. pneumoniae:* subject 1 in two of 46 weekly samples (in total: 5 clones); subject 2 in 13 of 49 samples (in total: 13 clones); subject 3 in 0 of 45 samples (in total: 0 clones); subject 4 in 15 of 48 samples (in total: 26 clones); subject 5 in 7 of 47 samples (in total: 10 clones); and subject 6 in 11 of 43 stool samples (in total: 17 clones) (Figure 1 and Table 1). Altogether, 80 *K. pneumoniae* isolates were retrieved from the 278 stool samples. Subject 3 was negative for *K. pneumoniae* colonization during the whole study period. The remaining five test persons were colonized with *K. pneumoniae* strains in samples accounting for a total of 2–15 weeks periods [mean: 8; median: 9] during the 1 year study period. No correlation between the number of *K. pneumoniae* positive stool samples and seasons could be observed (Figure 1).

**TABLE 1:** Typing results for isolates obtained from subjects 1–6 (no isolates were obtained from subject 3).

Proband	Sample ID	CW	ST	CT	wzc	wzi	K Serotype	O Serotype
1	510056-18	23/18	20	2,732	939	173	KL102	O2v2
	510059-18		277	2,733	914	97	KL46	O3b
	510076-18	30/18	4,099	2,743	940	114	KL111	O3b
	510077-18		306	2,744	12	11	KL11	O3/O3a
	510078-18		2,459	2,747	930	193	KL125	O3b
2	510005-18	16/18	1,265	2,688	nd	nd	KL33	O3b
	510017-18	17/18	152	2,695	22	87	KL163	O1v1
	510029-18	19/18	469	2,721	nd	75	KL105	O3b
	510034-18	21/18	632	2,724	nd	202	KL141	O4
	510035-18		611	2,725	930	193	KL125	O3b
	510046-18	22/18	301	2,729	42	42	KL42	O4
	510084-18	32/18	1,728	2,749	941	180	KL116	O2v1
	510087-18		1,758	2,750	28	187	KL27	O4
	510119-18	37/18	915	2,759	nd	150	KL107	OL101
	510126-18	38/18	915	2,759	nd	150	KL107	OL101
	510320-18	44/18	252	2,874	76	81	KL81	O1v2
	510340-18	45/18	322	2,876	18	50	KL17	O4
	510370-18	47/18	4,113	2,879	903	2	KL30	O3b
	510871-19	50/18	37	3,275	24	83	KL23	O2v2
	510902-19	8/19	322	2,876	18	50	KL17	O4
4	510929-19	11/18	20	3,312	29	84	KL28	O1v2
	510009-18	16/18	4,087	2,690	58	130	KL58	O3b
	510010-18		1,265	2,688	nd	nd	KL33	O3b
	510011-18		4,090	2,691	923	173	KL112	O2v2
	510021-18	17/18	105	2,696	939	383	KL102	O2v2
	510038-18	21/18	12	2,726	923	173	KL112	O2v2
	510041-18		461	2,727	nd	123	KL136	O2v1
	510049-18	22/18	632	2,724	nd	202	KL141	O4
	510050-18		34	2,731	23	27	KL37	O1v1
	510064-18	26/18	4,098	2,735	47	47	KL47	O1v1
	510065-18		704	2,739	32	31	Kl31	O1v1
	510066-18		492	2,737	15	23	KL14	O3b
	510067-18		530	2,736	54	115	KL54	O1v2
	510088-18	32/18	1,758	2,750	28	187	KL27	O4
	510089-18		37	2,751	930	85	KL125	O3/O3a
	510090-18		45	2,753	62	149	KL62	O2v1
	510092-18	33/18	200	2,754	28	150	KL27	O3b
	510096-18	34/18	200	2,754	28	150	KL27	O3b
	510112-18	36/18	469	2,721	nd	75	KL105	O3b
	510113-18		4,102	2,758	29	84	KL28	O1v2
	510127-18	38/18	461	4,860	26	133	KL25	O1v1
	510128-18		101	2,765	921	29	KL106	O1v2
	510306-18	41/18	1,159	2,870	29	84	KL28	O2v1
	510324-18	45/18	643	2,891	39	160	KL39	O1v1
	510325-18		1,141	2,893	940	114	KL111	O3b
	510326-18		1,334	2,892	nd	nd	OCL6	O3/O3a
	510334-18		730	2,895	nd	356	KL117	O1/O2v2
	510851-19	49/18	913	3,060	940	16	KL111	O3b
	510880-19	6/19	200	3,277	23	385	KL22	O3b
5	510013-18	16/18	37	2,692	nd	123	KL136	O2v2
	510025-18	17/18	2,662	2,720	nd	163	KL161	O4
	510026-18		4,092	2,809	nd	356	KL117	O2v2
	510027-18		37	2,692	nd	123	KL136	O2v2
	510042-18	21/18	22	2,728	9	9	KL9	O2v2
	510052-18	22/18	22	2,728	9	9	KL9	O2v2
	510068-18	29/18	889	2,738	903	73	KL104	O1v1
	510069-18		299	2,740	7	7	KL7	O2v1
	510070-18		1,430	2,741	41	39	KL107	OL102
	510071-18		3,626	2,768	8	114	KL8	O3b
	510336-18	45/18	45	2,894	25	101	KL24	O2v1
	510884-19	6/18	34	3,278	940	114	KL111	O3b
6	510001-18	15/18	314	2,687	24	82	KL23	O2v2
	510003-18		2,426	2,686	nd	356	KL117	O2v2
	510030-18	20/18	253	2,722	919	50	KL15	O4
	510031-18		26	2723	44	267	KL142	O1v1
	510060-18	24/18	200	2,734	14	39	KL13	O3b
	510072-18	29/18	3,640	2,742	64	333	KL64	O1v1
	510366-18	46/18	192	2,877	940	113	KL111	O3b
	510378-18	47/18	192	2,877	940	113	KL111	O3b
	510386-18	48/18	1,487	2,883	nd	197	KL141	O4
	510836-18		200	3,239	932	354	KL114	O3b
	510894-19	7/19	4121	3450	921	29	KL106	O2v2
	510919-19	10/19	279	3280	937	415	KL151	O5
	510920-19		4,122	3,281	32	31	KL31	O1v1
	510931-19	12/19	611	3,313	45	9	KL45	O2v2
	510939-19	13/19	1,426	3,315	21	177	KL20	O3/O3a
	510940-19		788	3,316	914	26	KL46	O3b
	510941-19		1,825	3,314	nd	361	KL126	OL101
	510945-19		4,123	3,318	58	22	KL58	O3b
	510946-19		323	3,319	61	155	KL62	O5

*CW, calendar week of isolation; ST, sequence type; CT, complex type.*

**FIGURE 2:**
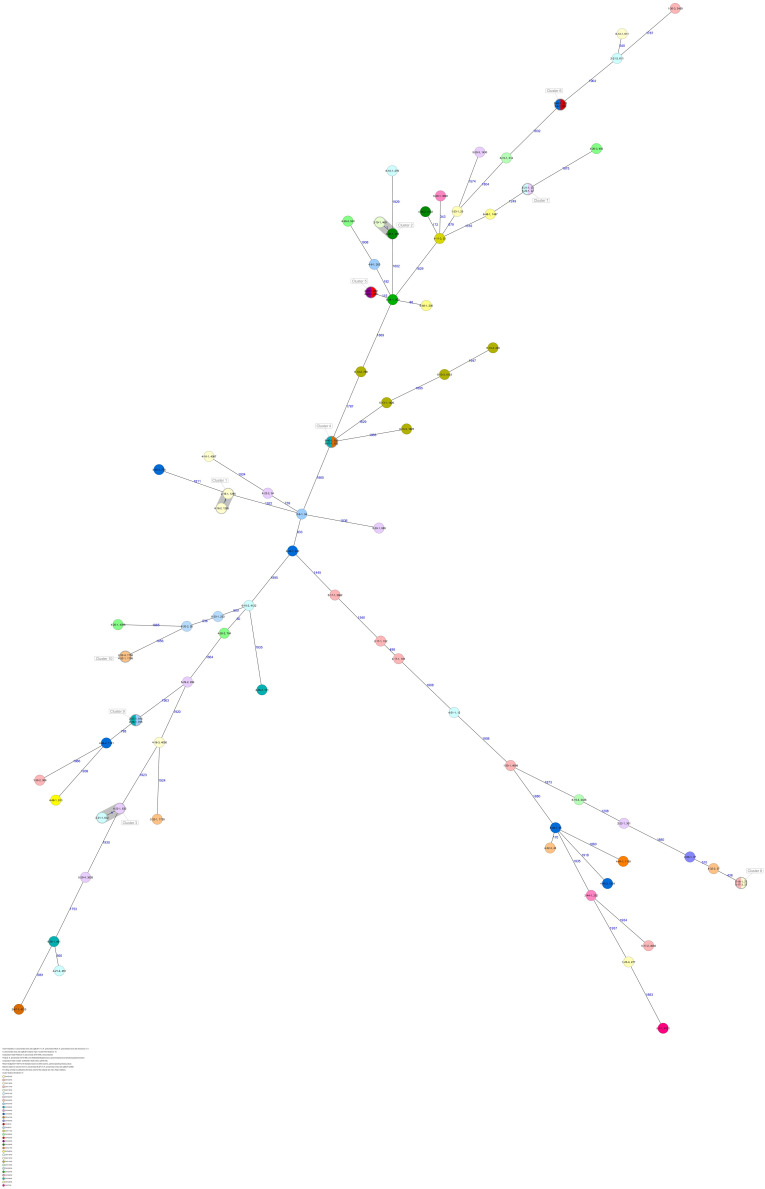
Minimum spanning tree (MST) based on cgMLST analysis of 80 *K. pneumoniae* isolates derived from subjects 1, 2, 4, 5, and 6. Numbers on connection lines represent allelic differences between isolates. Isolates are colored by sequence type (ST).

The 80 *K. pneumoniae* isolates were assigned to 60 different classical STs and 70 cgMLST complex types (CTs) (Table 1 and Figure 2). On average, all study isolates had 99.7% (98.6–100%) good core genome targets of the defined cgMLST scheme^6^. For nine isolates, which were obtained from the five colonized volunteers, new STs were determined and submitted to the *K. pneumoniae* MLST database^7^ : volunteer 1 (ST4099), volunteer 2 (ST4133), volunteer 4 (ST4090, ST4098, ST4102), volunteer 5 (ST4092), and volunteer 6 (ST4121, ST4122, and ST4123) (Table 1 and Figure 1). Serotype analysis from WGS data identified 39 *wzc* and 51 *wzi* alleles, 48 K serotypes, and 11 O serotypes (Table 1 and Supplementary Table 1). Eighteen isolates had no *wzc*, and in addition, three of these had no *wzi* either. Among the 48 K serotypes 22 had low or non-match confidence as defined by Kaptive-web (Supplementary Table 1). As shown above, inter- and intra-proband strain diversity was high with 60 different STs and 70 different CTs among 80 isolates. The volunteers were colonized with strains belonging to the same STs (ST20, ST34, ST37, ST45, and ST200) several times during the study period (Figures 1, 2 and Table 1). CgMLST analysis revealed an inter-patient core genome diversity of strains with the same ST from 88 to 679 allelic differences. Subjects 2 and 4 shared four strains with identical STs, CTs, and K serotypes: ST1265/CT2688/K33 isolates (Figure 2, cluster 1) were collected in CW16/18 and differed by one allele in their cgMLST (both volunteers had the same meal in the same restaurant the day before sampling); ST632/CT2724/K141 strains (Figure 2, cluster 3) were collected in CW21/18 and CW22/18 and differed by four alleles (both volunteers had the same meal at a birthday party in CW21); ST1758/CT2750/K27 strains (Figure 2, cluster 10) were collected in CW32/18 and shared the identical set of cgMLST alleles (both volunteers had the same meal at a birthday party in CW31); and ST469/CT2721/K105 strains (Figure 2, cluster 2) were collected in an interval of 17 CWs (CW19/18 and CW36/18) showing one allelic difference (no correlation detectable).

**FIGURE 3:**
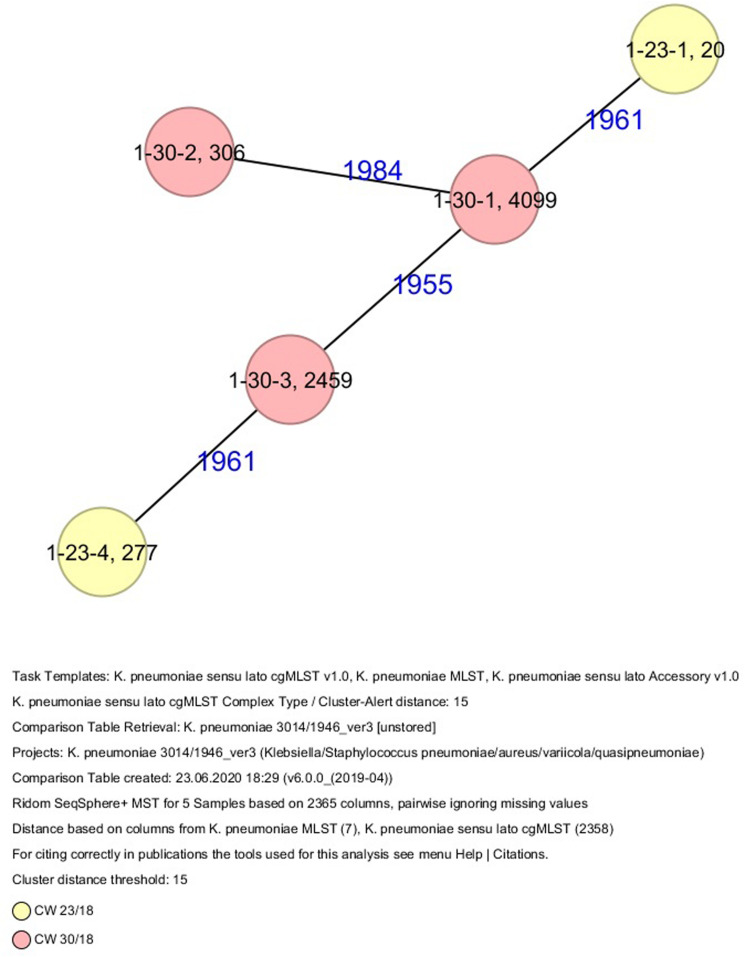
Minimum spanning tree (MST) based on cgMLST analysis of five *K. pneumoniae* isolates derived from subject 1. Numbers on connection lines represent allelic differences between isolates. Isolates are colored by date of isolation.

**FIGURE 4:**
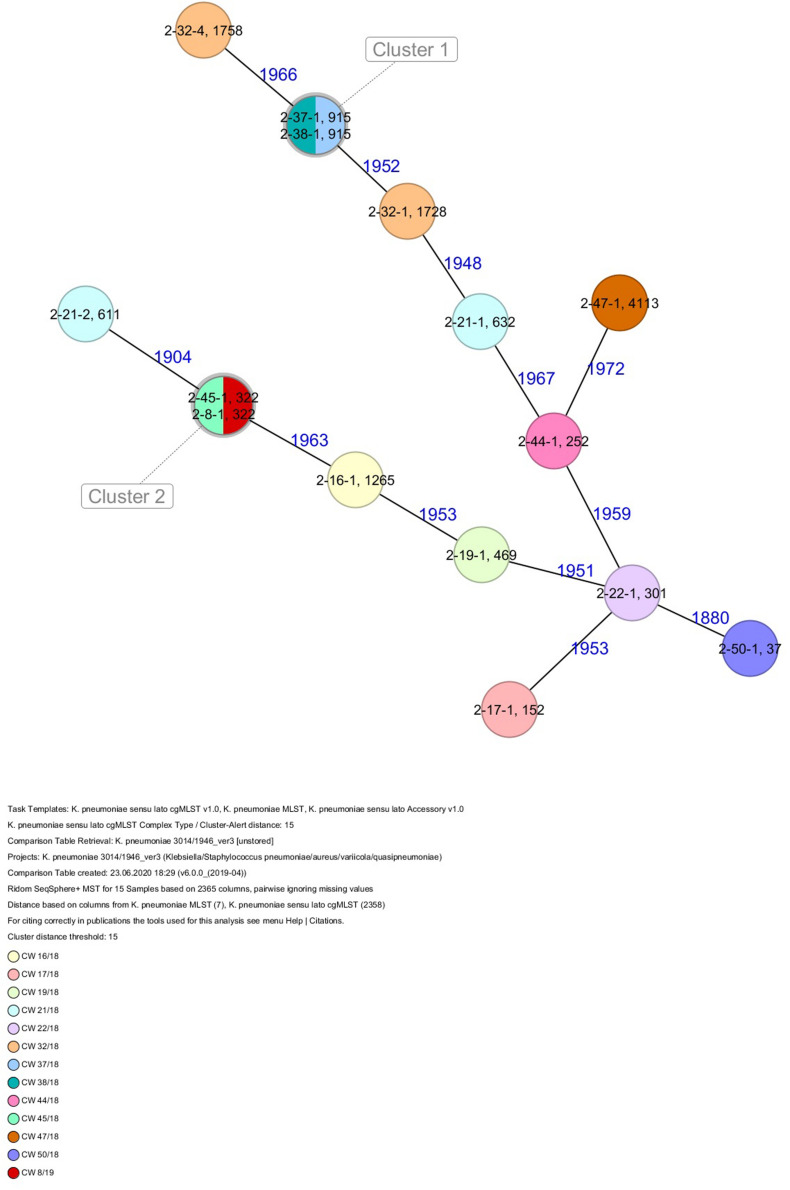
Minimum spanning tree (MST) based on cgMLST analysis of 15 *K. pneumoniae* isolates derived from subject 2. Numbers on connection lines represent allelic differences between isolates. Isolates are colored by date of isolation.

**FIGURE 5:**
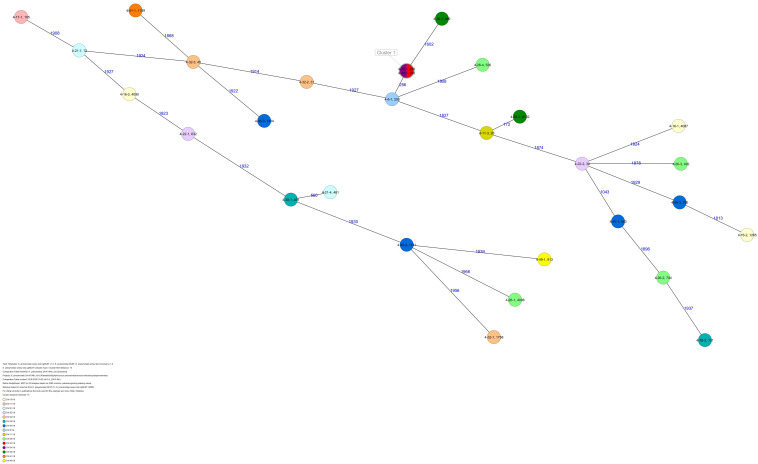
Minimum spanning tree (MST) based on cgMLST analysis of 29 *K. pneumoniae* isolates derived from subject 4. Numbers on connection lines represent allelic differences between isolates. Isolates are colored by date of isolation.

**FIGURE 6:**
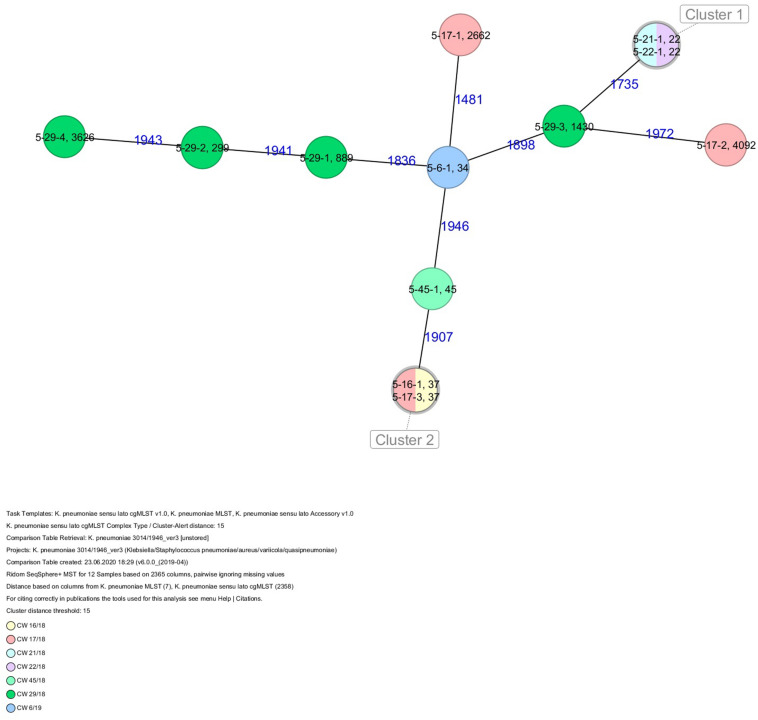
Minimum spanning tree (MST) based on cgMLST analysis of 12 *K. pneumoniae* isolates derived from subject 5. Numbers on connection lines represent allelic differences between isolates. Isolates are colored by date of isolation.

All five isolates of proband 1, which were derived from two stool samples, were unrelated as determined by ST, *wzc* and *wzi* allele typing, serotyping, and cgMLST analysis (Figures 2, 3 and Table 1). From proband 2, 15 isolates were cultured and assigned to 13 different STs and 13 different K serotypes. The volunteer was colonized with an ST915/CT2759/K107 isolate for two consecutive CWs (CW37/18-38/18) and with an ST322/CT2876/K17 isolate in CW45/18 and again 15 weeks later in CW8/19 (Figures 1, 4). Volunteer 2 was colonized with two different clones in CW21/18 and CW32/18 (Figures 1, 2, 4). From volunteer 4, 21 isolates were obtained and assigned to 26 different STs, 28 different CTs, and 23 K serotypes (Table 1 and Figure 5). Subject 4 was colonized for two consecutive CWs (CW33/18-34/18) with the same isolate (ST200/CT3277/K27) and was again colonized in CW 6/19 with an ST200/K27 isolate with CT2754 differing by 256 core genome alleles from isolate ST200/CT3277/K27. Later on, in CW 6/19, volunteer 4 was colonized with two different *K. pneumoniae* types in CW21/18, CW36/18, and CW38/18; with three different clones in CW16/18, CW22/18, and CW32/18. In CW26/18 and CW45/18, volunteer 4 was colonized with four different *K. pneumoniae* types (Figure 1 and Table 1). Subject 5 was colonized with 12 isolates that could be assigned to 10 different STs and 10 different K serotypes. Volunteer 5 was colonized with two identical isolates (ST22 and ST37) for two consecutive CWs (Figure 1 and Table 1). In CW17/18 and CW29/18, subject 5 was concurrently colonized with three and four respective different *K. pneumoniae* strains (Figure 1 and Table 1). Volunteer 6 was colonized by a total of 19 isolates, which were assigned to 17 different STs and 19 different K serotypes (Table 1). Volunteer 6 was colonized with an ST192 isolate for two consecutive CWs (CW46/18–47/18) (Figure 1 and Table 1). The other isolates differed by a maximum of 2012 alleles in their cgMLST (Figure 6). The volunteer was colonized with two different *K. pneumoniae* strains in CW15/18, CW20/18, CW48/18, and CW10/19 (Figure 1 and Table 1). In CW13/19, the volunteer was simultaneously colonized with five different K. *pneumoniae* strains (Figure 1 and Table 1).

**FIGURE 7:**
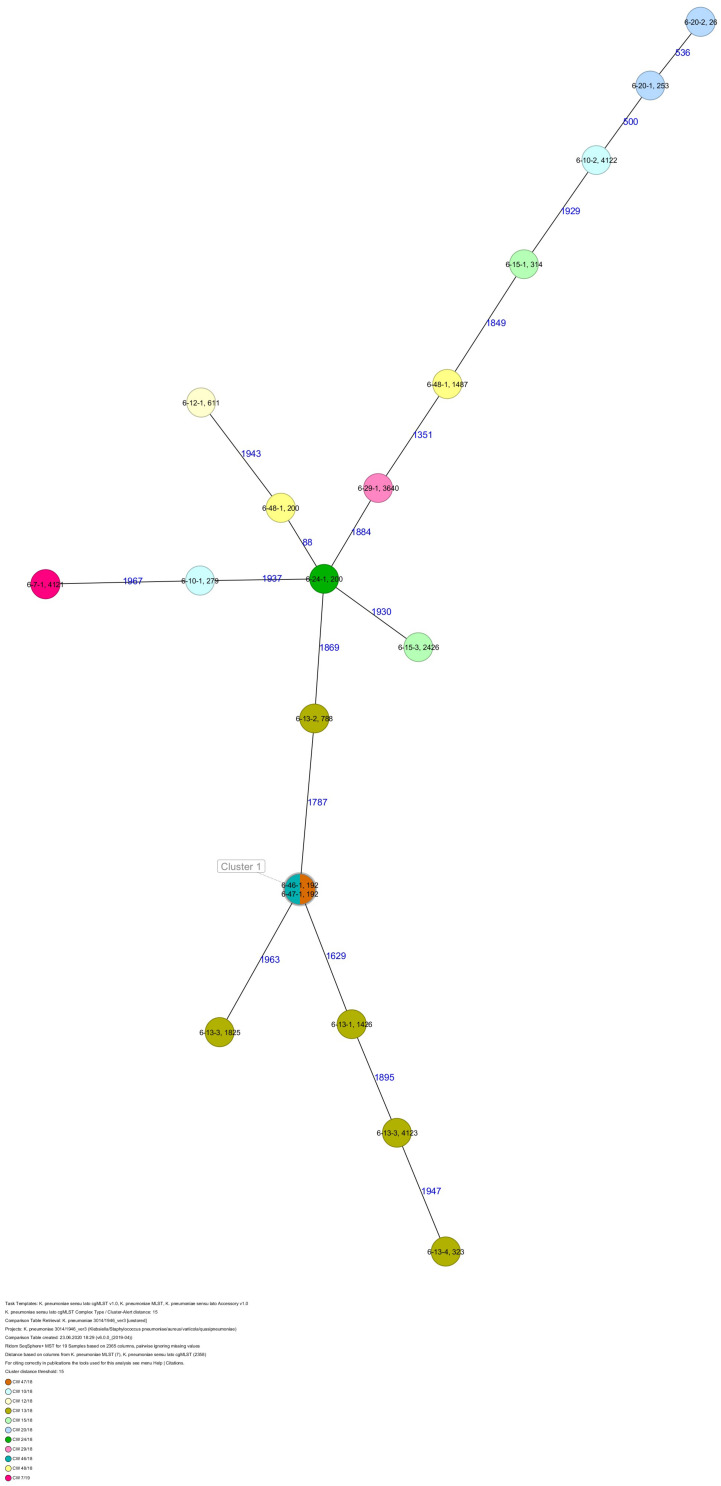
Minimum spanning tree (MST) based on cgMLST analysis of 19 *K. pneumoniae* isolates derived from subject 6. Numbers on connection lines represent allelic differences between isolates. Isolates are colored by date of isolation.

Strains with capsule types K20, K47, and STs ST20, ST37, ST45, ST101, ST152, ST323, and ST1265 (a single locus variant of hvKp ST23), which have been linked to high-risk clones (Huynh et al., 2020), were obtained several times (Table 1). Isolates with these capsule types or STs carried neither the characteristic hvKp virulence gene repertoire (Table 2 and Supplementary Table 1) nor the typical virulence plasmids (Supplementary Table 1).

**TABLE 2:** Virulence genes detected in *K. pneumonia* isolates of Probands 1–2 and 4–6.

Proband	Sample ID	ST	kfu	kvgA	fyuA	irp	mrk	ybt	Other genes
1	510056-18	20					A,B,D,F,H,I,J		
	510059-18	277					A,B,D,F,H,I,J		
	510076-18	4,099					A,B,D,F,H,I,J		
	510077-18	306					A,B,D,F,H,I,J		allD, allS, arcC, glxR
	510078-18	2,459					A,B,D,F,H,I,J		
2	510005-18	1,265					A,B,C,D,F,H,I,J		
	510017-18	152					A,F,H,I		
	510029-18	469					A,B,C,D,F,H,I,J		
	510034-18	632	A,C				A,B,C,D,F,H,I,J		
	510035-18	611					A,B,C,D,F,H,I,J		
	510046-18	301					A,B,C,D,F,H,I,J	A,Q,S,T,X	
	510084-18	1,728	A,C				A,B,C,D,F,H,I,J		
	510087-18	1,758					A,B,C,D,F,H,I,J		
	510119-18	915	A,C				A,B,C,D,F,H,I,J		
	510126-18	915	A,C				A,B,C,D,F,H,I,J		
	510320-18	252					A,D,F,H,I,J		
	510340-18	322					A,B,C,D,F,H,I,J		
	510370-18	4,113					A,B,C,D,F,H,I,J		mceA–E, mceG–J
	510871-19	37					A,B,C,D,F,H,I,J		
	510902-19	322					A,B,C,D,F,H,I,J		
4	510929-19	20				1,2	H,I,J	A,E,P,Q,S,T,U,X	
	510009-18	4,087					A,B,C,D,F,H,I,J		
	510010-18	1,265					A,B,C,D,F,H,I,J		
	510011-18	4,090					A,B,C,D,F,H,I,J		
	510021-18	105					A,B,C,D,F,H,I,J	A,E,P,Q,S,T,U,X	mceA–E, mceG–J
	510038-18	12					A,H,I,J		
	510041-18	461	A,B,C				C,D,F,H,I,J		
	510049-18	632	A,B,C				A,B,C,D,F,H,I,J		
	510050-18	34					A,H,I,J		
	510064-18	4,098	A,B,C				C,D,F,H,I,J		
	510065-18	704				1,2	B,C,H,I	A,E,P,Q,S,T,U,X	
	510066-18	492					A,B,C,D,F,H,I,J		
	510067-18	530					A,B,C,D,F,H,I,J		
	510088-18	1,758					A,B,C,D,F,H,I,J		
	510089-18	37					A,B,C,D,F,H,I,J		
	510090-18	45				1,2	A,B,C,D,H,I,J	A,E,P,Q,S,T,U,X	
	510092-18	200					A,B,C,D,F,H,I,J		
	510096-18	200					A,B,C,D,F,H,I,J		
	510112-18	469					A,B,C,D,F,H,I,J		
	510113-18	4,102					A,B,C,D,F,H,I,J		
	510127-18	461	A,B,C				A,H,I,J		
	510128-18	101	A,B,C				A,B,C,D,F,H,I,J		
	510306-18	1,159					A,B,C,D,F,H,I,J		
	510324-18	643					A,H,I,J		
	510325-18	1,141	A,B,C				A,B,C,D,F,H,I,J		
	510326-18	1,334					A,B,C,D,F,H,I,J		
	510334-18	730					A,D,H,I		
	510851-19	913	A,B,C				A,B,C,D,F,H,I,J		mceA–E, mceG–J
	510880-19	200					A,B,C,D,F,H,I,J		
5	510013-18	37					A,B,C,D,F,H,I,J		
	510025-18	2,662					H,I		
	510026-18	4,092					A,B,C,D,F,H,I,J		
	510027-18	37					A,B,C,D,F,H,I,J		
	510042-18	22					A,B,C,D,F,H,I,J		
	510052-18	22					A,B,C,D,F,H,I,J		
	510068-18	889	A,B,C				A,B,C,D,F,H,I,J		allD, KP1_1371
	510069-18	299					B,H,I,J		
	510070-18	1,430					A,B,F,H,I,J		
	510071-18	3,626	A,B,C				C,D,H,I		
	510336-18	45				1,2	A,B,C,D,H,I,J	A,E,P,Q,S,T,U,X	
	510884-19	34					A,B,C,D,F,H,I,J		
6	510919-19	279				1,2	A,B,C,D,H,I,J	A,E,P,Q,S,T,U,X	
	510920-19	4,122				1,2	A,B,D,H,I,J	A,E,P,Q,S,T,U,X	
	510931-19	611				1,2	A,B,C,D,H,I,J	A,E,P,Q,S,T,U,X	
	510939-19	1,426					A,B,C,D,F,H,I,J		
	510940-19	788					A,B,C,D,F,H,I,J		
	510941-19	1,825					A,B,C,D,F,H,I,J		
	510945-19	4,123					A,B,C,D,F,H,I,J		
	510946-19	323					A,B,C,D,F,H,I,J		
	510001-18	314					A,B,C,D,F,H,I,J		
	510003-18	2,426					A,B,C,D,F,H,I,J		
	510030-18	253					H,I		
	510031-18	26					A,B,C,D,F,H,I,J		
	510060-18	200					A,B,C,D,F,H,I,J		
	510072-18	3,640					A,F,H,I,J		
	510366-18	192				1,2	A,B,C,D,H,I,J	A,E,P,Q,S,T,U,X	
	510378-18	192				1,2	A,B,C,D,H,I,J	A,E,P,Q,S,T,U,X	
	510386-18	1,487					A,B,C,D,F,H,I,J		
	510836-19	200					A,C,D,H,I		allB, allD, allS, KP1-1371, hyi, ybbW
	510894-19	4,121					A,B,C,D,F,H,I,J		

The analysis for the presence of virulence genes identified a total of 42 different virulence genes among all 80 isolates (Table 2). Genes belonging to the type 3 fimbrial gene cluster (*mrk*) were present in all isolates. Three isolates [3.75%, ST306 (volunteer 1), ST889 (volunteer 5), and ST200 (volunteer 6)] harbored additional genes associated with the allantoin metabolism (*all, glx, hyi*), 14 isolates (17.5%, volunteers 2, 4, and 5) harbored genes encoding an intestinal colonization factor (*kfu*), 10 isolates (12.5%, volunteers 2 and 5) carried genes that contribute to capsule formation (*kvg*), 3 isolates (3.75%, volunteers 2 and 4) harbored genes belonging to the mammalian cell entry (*mce*) gene cluster, and 10 isolates (12.5%, volunteers 2, 4, 5, and 6) had genes encoding yersiniabactin (*ybt*) (Table 2 and Supplementary Table 1).

Phenotypic testing of isolates for their ESBL producing capacity revealed that all isolates were ESBL negative. The analysis for antibiotic resistance genes revealed 25 genes among all 80 isolates in total (Table 3 and Supplementary Table 1). Out of these 25 resistance genes, 13 were present in all investigated isolates: *baeR* (antibiotic efflux), CRP (antibiotic efflux), *emrB* (antibiotic efflux), *emrR* (antibiotic efflux), *fosA* (antibiotic efflux), *marA* (antibiotic efflux), *marR* (antibiotic efflux), *msbA* (antibiotic efflux), H-NS (antibiotic efflux), *oqxA/B* (antibiotic efflux), EF-Tu (R234F, antibiotic target alteration), *uhpT* (E350Q, antibiotic target alteration), and *bla*_*SHV*_ (antibiotic inactivation). The isolates carried 18 different SHV variants (Table 3 and Supplementary Table 1).

**TABLE 3:** Resistance genes/mechanisms, SHV variants and ESBL phenotypes detected in *K. pneumoniae* isolates of this study.

Proband	Sample ID	ST	emr	FosA	Kp-acrA	vgaC	SHV	Other genes	ESBL phenotypic testing
1	510056-18	20	B,R	6			1		S
	510059-18	277	B,R	6			27		S
	510076-18	4,099	B,R	6			1		nd
	510077-18	306	B,R	6			1		nd
	510078-18	2,459	B,R	6			1		nd
2	510005-18	1,265	B,R	5			36		nd
	510017-18	152	B,R	6			1		nd
	510029-18	469	B,R	5			11		nd
	510034-18	632	B,R	6			108		S
	510035-18	611	B,R	6			27		S
	510046-18	301	B,R	6			27		S
	510084-18	1,728	B,R	6			11		nd
	510087-18	1,758	B,R	6			1		nd
	510119-18	915	B,R	6			11		nd
	510126-18	915	B,R	6			11		nd
	510320-18	252	B,R	6			1		nd
	510340-18	322	B,R	6			11		nd
	510370-18	4,113	B,R	5			142		nd
	510871-19	37	B,R	5			11		S
	510902-19	322	B,R	6			11		nd
4	510929-19	20	B,R	5			187		nd
	510009-18	4,087	B,R	6			11		nd
	510010-18	1,265	B,R	5			36		nd
	510011-18	4,090	B,R	6			1		nd
	510021-18	105	B,R	6			1		nd
	510038-18	12	B,R	6			11		nd
	510041-18	461	B,R	5			1		nd
	510049-18	632	B,R	6			108		nd
	510050-18	34	B,R	5			71		nd
	510064-18	4,098	B,R	5			75		nd
	510065-18	704	B,R	5			36		nd
	510066-18	492	B,R	5			11		nd
	510067-18	530	B,R	5			1		nd
	510088-18	1,758	B,R	6			1		nd
	510089-18	37	B,R	6			51		nd
	510090-18	45	B,R	6			1		nd
	510092-18	200	B,R	5			1		nd
	510096-18	200	B,R	5			1		nd
	510112-18	469	B,R	5			11		nd
	510113-18	4,102	B,R	5			187		nd
	510127-18	461	B,R	5			1		nd
	510128-18	101	B,R	6			1		nd
	510306-18	1,159	B,R	6			11		nd
	510324-18	643	B,R	5			26		nd
	510325-18	1,141	B,R	6,7			11		nd
	510326-18	1,334	B,R	6			164		nd
	510334-18	730	B,R	5			1		nd
	510851-19	913	B,R	6,7			28	oprN, mexF	nd
	510880-19	200	B,R	5			1		nd
5	510013-18	37	B,R	5			11		nd
	510025-18	2,662	B,R	6			26		nd
	510026-18	4,092	B,R	6			1	APH(3?)-lb, APH(6)-ld	S
	510027-18	37	B,R	6			11		nd
	510042-18	22	B,R	6			1		nd
	510052-18	22	B,R	6			1		nd
	510068-18	889	B,R	6			108	qnrS	S
	510069-18	299	B,R	6			1		nd
	510070-18	1,430	B,R	6			26	tetD	nd
	510071-18	3,626	B,R	6			37	rpoB2	S
	510336-18	45	B,R	6			1		nd
	510884-19	34	B,R	6			50		nd
6	510919-19	279	B,R	6			11		nd
	510920-19	4,122	B,R	5			36		nd
	510931-19	611	B,R	6			27		S
	510939-19	1,426	B,R	6			11		nd
	510940-19	788	B,R	5			52	tetC	S
	510941-19	1,825	B,R	6			11		nd
	510945-19	4,123	B,R	5			11		nd
	510946-19	323	B,R	5			52	tetC	nd
	510001-18	314	B,R	6			1		nd
	510003-18	2,426	B,R	6			27		S
	510030-18	253	B,R	5			36		nd
	510031-18	26	B,R	5			36	acrA-MDR, APH(3”)-lb, acrD	S
	510060-18	200	B,R	5			1		nd
	510072-18	3,640	B,R	5			40		S
	510366-18	192	B,R	5			60		nd
	510378-18	192	B,R	5			60		nd
	510386-18	1,487	B,R	5			11		nd
	510836-19	200	B,R	5			1		nd
	510894-19	4,121	B,R	6			168	mfbA, SAT-2,tetA, tetR, aadA	S

*S, sensitive; nd, not done.*

The resistance genes *ompK37* (bacterial porin), *acrA* (antibiotic efflux), and *vgaC* (antibiotic target protection) were present in 97.5% (*n* = 78), 91.3% (*n* = 73), and 48.8% (*n* = 39) of all isolates.

Variants of the *tet* resistance gene [*tet*(A), *tet*(C), and *tet*(D)], which encode antibiotic efflux and confer tetracycline resistance, were present in isolates from subjects 5 (*n* = 1) and 6 (*n* = 4).

Resistance genes APH(3″)-Ib and APH(6)-Id, both conferring resistance to aminoglycoside antibiotics by antibiotic inactivation, were detected in two isolates (ST4092 and ST26) from subjects 5 and 6 in different CWs. Three resistance genes were detected exclusively in isolates from subject 6: *aadA* (antibiotic inactivation), *mfpA* (antibiotic target protection), and *sat-2* (antibiotic inactivation) (Table 3). Two determinants conferring resistance were present in two isolates only from subject 5: *qnrS2* (antibiotic target protection) and *rpoB2* (antibiotic target alteration). Two genes (*mexF*, *oprN*), encoding antibiotic efflux, were only present in two isolates from subject 4 (Table 3).

The detection of plasmids via the PlasmidFinder tool revealed that 65 (81.3%) isolates carried plasmids. Among these 65 isolates, 16 different plasmid types: [Col(IMGS31) (*n* = 1); Col440I (21); Col440II (10); FII(pBK30683) (1); IncFIA(HI1) (6); IncFIB(K) (41); IncFIB(Mar) (3); IncFIB(pKPHS1) (5); IncFIB(pQil) (1); IncFII (7); IncFII(K) (28); IncFII(Yp) (2); IncHI1B (7); IncN3 (1); IncR (23); IncX3 (1)] were detected and the number of plasmids per isolate varied from 1 to 5 plasmids (Supplementary Table 1). No plasmids described as hvKp specific, i.e., pK2044 and pLVPK, were detected (Supplementary Table 1).

## Discussion

Recent studies have shown that gastrointestinal colonization with *K. pneumoniae* is a common and significant reservoir for the transmission and subsequent infection of patients (Martin et al., 2016; Dorman and Short, 2017; Gorrie et al., 2017).

In our study, *K. pneumoniae* was found in 0.0–31.3% (mean 17.2%) of stool samples tested. This is lower than in previous studies with a colonization rate of 37.5% (Marques et al., 2019) and 55.9% (Huynh et al., 2020) but is in concordance with other studies reporting 4–10% colonization rates for test subjects (Choby et al., 2020). In contrast to other studies, where an increased fecal carriage rate during the summer was reported (Anderson et al., 2008), no such seasonal correlation could be observed in our study. It is of interest that one individual remained *K. pneumoniae* free during the entire 1 year study period. An explanation for this colonization failure might be a specific composition of the test persons’ microbiota that prevented *K. pneumoniae* from persisting in the gut, as has previously been shown in ICU patients (Collingwood et al., 2020). All other five participants in our study were colonized with *K. pneumoniae* strains in at least one of the weekly obtained samples, with individual stool samples yielding up to five different strains.

Colonization with multiple strains has already been reported in other studies (Marques et al., 2019). *K. pneumoniae* high-risk clonal lineages are either multidrug-resistant strains mainly causing severe infections in hospitals (Navon-Venezia et al., 2017) or are drug-susceptible hypervirulent strains (hvKp) causing infections in the community mainly in younger and healthy individuals (Paczosa and Mecsas, 2016). High-risk clonal lineages of the multidrug-resistant type exist worldwide and can be assigned to certain *K. pneumoniae* STs (Roe et al., 2019; Huynh et al., 2020). Although mainly found in hospitals, these clones can also colonize individuals outside hospitals (Holt et al., 2015). Since colonization is a potential reservoir for infection with *K. pneumoniae* strains (Gorrie et al., 2017), investigation of the rates and duration of carriage is of importance to assess the potential risk for that community. In our study, the diversity of isolates colonizing the test persons was high and colonization with specific strains occurred for a maximum of two consecutive weeks. Also based on the finding that two individuals who regularly ate meals together were colonized several times with identical strains, we hypothesize that the high diversity of isolates in our study is due to the consumption of contaminated food; food as a source of *K. pneumoniae* carriage has been previously described (Huynh et al., 2020; Koliada et al., 2020). The observed colonization of healthy individuals with diverse strains but for short time periods is in contrast to the situation in hospitals where patients are colonized over long periods of time with specific resistant clones due to treatment with antibiotics (Martin et al., 2016). In our study, no multidrug-resistant *K. pneumoniae* isolates were found, which is concordant to recent studies on healthy individuals without reported use of antibiotics (Marques et al., 2019; Huynh et al., 2020). In total, 25 resistance genes—mainly genes encoding for efflux pumps—were found among all 80 *K. pneumoniae* isolates. All isolates carried SHV beta-lacatamases.

In contrast to Asian countries, where the prevalence of hvKp is high (Chung et al., 2012), no hvKp isolates were detected in our study. None of the isolates carried the unique hvKp virulence gene repertoire or virulence plasmids in combination with hvKp-specific K serotypes or STs (Struve et al., 2015; Gu et al., 2018; Choby et al., 2020; Yang et al., 2020). However, some isolates carried single hvKp-specific virulence genes. Two isolates, ST306/K11/O3/O3a and ST1487/K141 (non-match confidence)/O4, carried the allantoinase gene cluster, reported to be exclusively present in hvKp CC23 and ST25 clonal lineages (Struve et al., 2015). Virulence factors *kfu* or the yersiniabactin gene cluster *ybt* were found in 10 isolates with diverse STs and K serotypes. Two patients were colonized with ST20 isolates, which have been described as an international outbreak clone (Mavroidi et al., 2014; Yu et al., 2016; Patil et al., 2019). The isolates had different K serotypes and carried SHV-1 and SHV-187 in contrast to the outbreak strains described, which were SHV-5, NDM-1, OXA-48, and KPC-2 producers (Mavroidi et al., 2014; Yu et al., 2016; Patil et al., 2019).

In conclusion, our study revealed that fecal *K. pneumoniae* carriage is intermittent and of high clonal diversity. Colonization with specific strains could be observed for a maximum of only two consecutive calendar weeks. Two of the five colonized individuals—working colleagues having the same meals together several times—shared identical *K. pneumoniae* types four times during the study period pointing toward the potential role of food as a reservoir of *K. pneumoniae* for humans as also described recently (Huynh et al., 2020). In contrast to *E. coli*, which is a lifelong colonizer of the human gut (Palmer et al., 2007), *K. pneumoniae* seems unable to colonize a healthy human permanently.

## Data Availability Statement

The whole genome sequencing datasets generated for this study can be found in the DDBJ/EMBL/GenBank; accession PRJNA663884.

## Ethics Statement

The studies involving human participants were reviewed and approved by Dr. Michael Tamchina, Co-chair of the Ethics committee of the city of Vienna, Thomas Klestil Platz 8, 1030 Vienna, michael.tamchina@wien.gv.at. The patients/participants provided their written informed consent to participate in this study.

## Author Contributions

SL, KH, CS, BS, FA, and WR: conceptualization. SL, KH, ASc, CS, ASt, CV, and SP-A: methodology. SL and WR: software analysis. KH, ASc, CS, CV, and FA: resources. SL, KH, ASc, and WR: data curation. SL, KH, BS, FA and WR: writing–original draft preparation. SL, KH, ASc, BS, FA, and WR: writing–review and editing. CS, BS, and FA: project administration. BS and FA: funding acquisition. All authors contributed to the article and approved the submitted version.

## Conflict of Interest

The authors declare that the research was conducted in the absence of any commercial or financial relationships that could be construed as a potential conflict of interest.

## Footnotes

1 https://www.cgmlst.org/ncs/schema/2187931/

2 http://bigsdb.pasteur.fr/

3 https://kaptive-web.erc.monash.edu

4 http://www.genomicepidemiology.org/

5 http://bigsdb.pasteur.fr/klebsiella

6 http://www.cgmlst.org

7 http://www.pasteur.fr/mlst/
